# Vestibular dysfunction in pediatric patients with cochlear implantation: A systematic review and meta-analysis

**DOI:** 10.3389/fneur.2022.996580

**Published:** 2022-10-17

**Authors:** Qiong Wu, Qin Zhang, Qianwen Xiao, Yuzhong Zhang, Zichen Chen, Shuyun Liu, Xueyan Wang, Yong Xu, Xin-Da Xu, Jingrong Lv, Yulian Jin, Jun Yang, Qing Zhang

**Affiliations:** ^1^Department of Otolaryngology Head and Neck Surgery, Xinhua Hospital, Shanghai Jiaotong University School of Medicine, Shanghai, China; ^2^Ear Institute, Shanghai Jiaotong University School of Medicine, Shanghai, China; ^3^Shanghai Key Laboratory of Translational Medicine in Ear and Nose Diseases, Shanghai, China; ^4^Department of Otolaryngology Head and Neck Surgery, Second Affiliated Hospital of Xi'an Jiaotong University, Xi'an, Shanxi, China; ^5^Department of Otolaryngology Head and Neck Surgery, The Affiliated Hospital of Southwest Medical University, Luzhou, Sichuan, China; ^6^Department of Otolaryngology Head and Neck Surgery, The Affiliated Hospital of Yanbian University, Yanji, Jilin, China; ^7^Department of Otolaryngology, Eye and ENT Hospital, Fudan University, Shanghai, China; ^8^Diagnosis and Treatment Center of Hearing Impairment and Vertigo, Xinhua Hospital, Shanghai Jiaotong University School of Medicine, Shanghai, China

**Keywords:** cochlear implantation, vestibular function test, vestibular-evoked myogenic potentials, vestibular disorders, pediatric patients

## Abstract

**Objective:**

Vestibular dysfunction may delay the achievement of balance and perception milestones in pediatric patients after cochlear implantation (CIM).

**Methods:**

A strategic literature search was done following Preferred Reporting Items for Systematic Reviews and Meta-Analyses (PRISMA) guidelines. We searched the PubMed, Medline, Embase, Web of Science, and Cochrane Library databases from inception to July 2022. Studies were included on the otoliths, semicircular canals, and balance function changes in children after CIM. Two reviewers independently assessed the level of evidence, methodological limitations, risk of bias, and characteristics of the cases. Matched pre- and postoperative vestibular functional test data, including ocular and cervical vestibular-evoked myogenic potential (oVEMP and cVEMP), caloric test, video head impulse test (vHIT), and Bruininks-Oseretsky Test 2 (BOT-2), were used to calculate the relative risk of vestibular disorders. Subgroup analyses were performed according to surgical approach, CIM device status, and etiology.

**Results:**

Twenty studies that met the inclusion criteria were selected for the meta-analysis. We observed significant vestibular dysfunction in pediatric patients with CIM. The results showed a statistically significant increase in abnormal cVEMP response (RR = 2.20, 95% CI = 1.87, 2.58, *P* < 0.0001), abnormal oVEMP response (RR = 2.10, 95% CI = 1.50, 2.94, *P* < 0.0001), and abnormal caloric test results (RR = 1.62, 95% CI = 1.20, 2.19, *P* = 0.0018) after implantation. Statistically significant differences were not found in the vHIT test results of all three semicircular canals before and after the operation (*P* > 0.05). Regarding static and dynamic balance, we found significantly poorer BOT-2 scores in children with CIM than in the normal group (mean difference = −7.26, 95% CI = −10.82, −3.70, *P* < 0.0001).

**Conclusion:**

The results showed that vestibular dysfunction might occur after CIM in pediatric patients. Some children experience difficulties with postural control and balance. Our results suggest that a comprehensive evaluation of vestibular function should be performed before and after CIM.

## Introduction

Cochlear implantation (CIM) is the gold standard for treating severe to profound unilateral or bilateral sensorineural hearing loss (SNHL) in pediatric patients. CIM significantly improves hearing levels, speech intelligibility, and sound localization in quiet and noisy environments (1, 2). Thus, implantation should be performed in children with congenital SNHL as early as possible once confirmatory diagnostics are reliably completed.

Although CIM is a safe and conventional surgical procedure, the possible consequences and risks posed by CIM should be evaluated (3). As the importance of vestibular preservation has been widely acknowledged, an increasing number of studies have found that CIM can increase the risk of vestibular dysfunction (4–12). Congenital or acquired vestibular dysfunction in infants and children normally leads to impaired postural control, gait disturbances, and delayed locomotion development (13–15). Thus, the development, status, and damage to vestibular function in pediatric patients after CIM have been widely studied by researchers.

The vestibular function can be measured based on the cervical vestibular-evoked myogenic potential (cVEMP), ocular VEMP (oVEMP), caloric test, and video head impulse test (vHIT) (16, 17), and the symptoms of vestibular dysfunction commonly manifest as dizziness or postural imbalance (18).

Vestibular ramifications in adults after CIM have been documented (19–21). In a meta-analysis, Ibrahim et al. (22) observed that CIM surgery had a significant negative effect on the results of cVEMP and caloric tests, while Hänsel et al. (23) reported a notable increase in postoperative subjective vertigo and vestibular dysfunction. Nevertheless, assessing vestibular function in children seems difficult due to the difficulty and non-compliance in testing pediatric patients and the lack of available equipment. A few related studies of pre- and postoperative vestibular function focused on CIM in children. A recent systematic review showed subjective and objective vestibular changes following pediatric CIM. Due to the lack of quantitative data in some vestibular and balance function measurements, we only detected vestibular function by analyzing cVEMP and caloric test results (24).

The innovation of the current meta-analysis is that it demonstrated the difference in vestibular function between the pre- and postoperative statuses of pediatric patients by comprehensively comparing various vestibular function tests, including the cVEMP, oVEMP, caloric, and vHIT tests. We also evaluated the balance function in children using the Bruininks-Oseretsky Test of Motor Proficiency 2 (BOT-2) balance subtest. Thus, we aimed to systematically clarify the alterations in vestibular function following CIM in pediatric patients and the factors that may influence these results.

## Materials and methods

### Data retrieval

The specifications for this systematic review were formulated in accordance with the Preferred Reporting Items for Systematic Reviews and Meta-analysis (PRISMA) statement (25). The PRISMA checklist is shown in Supplementary Table S1.

### Search strategy

Online databases, including PubMed, Medline, Embase, Web of Science, and the Cochrane Library, were searched by two independent authors (QW and QZ). Observational cohort studies of vestibular function changes after CIM were retrieved from the establishment of the database until July 9, 2022. Specific keywords consisted of Medical Subject Headings (MeSH) and free-text terms: “vestibular system,” “vestibular evoked myogenic potentials,” “vestibular function test,” “vestibular diseases,” “vertigo,” “vestibular, labyrinth,” “proprioception,” “reflex, vestibular-ocular,” “saccule and utricle,” “vestibular disorders,” “vestibular dysfunction,” “vestibular impairment,” “cochlear implants” or “cochlear implantation,” and “all child.” In addition, correlative references from eligible publications were examined. The disagreements regarding the exclusion or inclusion of specific studies were resolved by the third author (QZ) after discussion with all the research group members.

### Eligibility criteria

We systematically retrieved the literature using the PICOS model (Population, Intervention, Comparison, Outcomes, Study design) (Table 1).

**Table 1:** PICOS model.

**Population**	**Pediatric patients with unilateral or bilateral sensorineural hearing loss**
Intervention	After CIM
Comparison	Before CIM
Outcomes	The results of cVEMP, oVEMP, caloric tests, vHIT, and BOT-2 balance subtest
Study design	Observational studies (prospective and retrospective cohort studies)

#### Inclusion criteria

(1) Prospective or retrospective cohort studies comparing vestibular function before and after CIM;
(2) Studies including pediatric patients (age <18 years);
(3) Necessary results of various vestibular function tests are available in the manuscript, including the results of cVEMP, oVEMP, caloric, and vHIT tests;
(4) Studies reporting BOT-2 balance subtest results;
(5) Studies including children with unilateral or bilateral CIM regardless of the surgical method used;
(6) Selection of studies with the largest number of participants in the case of overlapping samples.

#### Exclusion criteria

(7) Studies not published in English;
(8) Studies that focused only on pre- or post-CIM;
(9) Case reports, editorials, and commentaries;
(10) Publications do not report appropriate data for performing a meta-analysis.

### Data extraction

Microsoft Excel (Microsoft, Redmond, WA, USA) was used to independently perform data extraction and literature screening by two researchers (QWX and SYL). Disagreements were resolved by cross-checking and discussion. The extracted data included (1) family name of the first author and publication year. (2) study design. (3) patient country. (4) sample size. (5) age of patients. (6) etiology of SNHL. (7) specific surgical measures for CIM. (8) unilateral or bilateral CIM. (9) time of vestibular function test postoperatively. (10) vestibular function test methods, and (11) references list. We evaluated the heterogeneity and external validity of the selected studies using this information.

### Quality assessment

The Newcastle-Ottawa scale, which is comprehensive and has been partially validated to assess the quality of observational research in meta-analyses, was used to estimate the quality of the included studies. The Newcastle-Ottawa scale is a checklist that evaluates the quality of literature based on three categories: selection (composed of four items with a maximum score of 4 points), comparability of the study groups (composed of one item with a maximum score of 2 points), and ascertainment of exposure or outcome of interest (composed of three items with a maximum score of 3 points). A “star system” (ranging from 0 to 9) has been developed for evaluation. A score of <7 was designated as low quality; higher scores indicated high-quality studies. Quality evaluations were performed independently by two authors (YZ and ZC). According to statistics, all the 20 documents included in the meta-analysis meet the conditions.

### Heterogeneity

Methodological and clinical heterogeneity were assessed by inspecting the characteristics of the studies, outcomes, the similarity between the types of participants, and interventions as specified in the inclusion criteria. The chi^2^ test and I^2^ statistic were used to evaluate statistical heterogeneity. I^2^ ≥ 50% indicated substantial heterogeneity, and the meta-analysis recommended the random-effects model. I^2^ <50% demonstrated notable homogeneity, and the fixed-effects model was used. Low, moderate, and significant heterogeneity were determined according to I^2^ values of 25, 50, and 75%, respectively. Sensitivity analysis was used to check whether any single study accounted for the heterogeneity.

### Data analyses

Major outcomes included differences in vestibular function test results between the pre- and postoperative periods in children with CIM. The results of the cVEMP, oVEMP, caloric, vHIT, and BOT-2 tests were examined as major parameters. For performing the meta-analysis, in the case of binary variables, we calculated the relative risk (RR) and 95% confidence interval (95% CI) as the effect size using the maximum likelihood method; for continuous variables, the effect size was measured using the mean difference and standardized mean difference in scores of the normal and CIM groups.

Regarding statistical analysis, all data processing and graph plotting in the meta-analysis were performed with R version 4.1.0 (R Foundation for Statistical Computing, Vienna, Austria), using the R-package (metagen). Statistical significance was set at *P* < 0.05.

## Results

### Literature search

**Figure 1:**
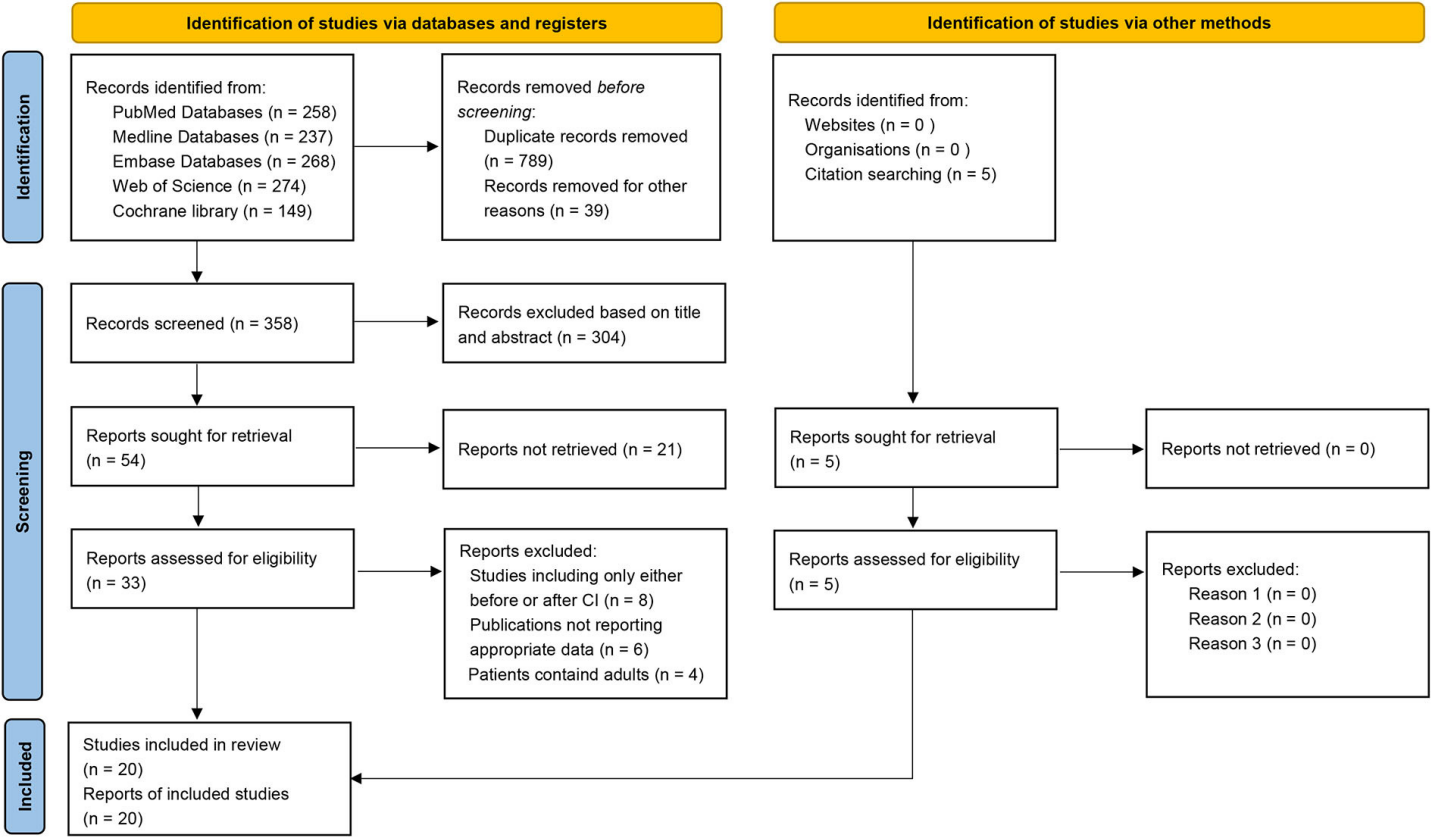
PRISMA flow diagram of the systematic literature search process.

The systematic review identified 1,186 studies *via* databases and registers. After manually removing 789 duplicate studies and 39 studies that were irrelevant to the subject, 358 records were screened. After title and abstract screening, 304 studies were excluded. The remaining 54 studies were retrieved for full-text appraisal. We eliminated 21 reports for which the full text was unavailable, eight studies that only considered the pre- or post-CIM period, six studies without appropriate data, and four studies with adults (age ≥ 18 years). After reviewing the research references, five additional studies were identified. Thus, 20 studies were finally selected for the meta-analysis (7, 15, 26–43) (Figure 1).

### Included study characteristics

The specific characteristics of the 20 selected studies are summarized in Table 2. Their publication dates ranged from 2006 to 2022. Five studies had unknown study designs, eight had a prospective study design, five had a retrospective study design, and two were only observational studies without a specific study design. Most of the 20 studies were performed in Asia (10 studies from China, Japan, India, and Iran, with a total of 299 patients), followed by North America (five studies from Canada and the USA; a total of 215 patients), Europe (four studies from France, Romania, Belgium, and Greece; a total of 131 patients), and Africa (one study from Egypt with 40 patients). The detailed etiologies of 687 patients (age range 1–18 years) are shown in Table 2. The surgical approach for electrode insertion was a round window (RW) or extended RW in 6 studies, cochleostomy in four studies, both RW and cochleostomy in two studies, and no specified approach in eight studies. In addition, the study also determined the methods of vestibular function tests, the implanted side, and the time of postoperative vestibular function tests.

**Table 2:** Study demographics.

**Study**	**Design**	**Country**	**Sample size**	**Age**	**Etiology**	**Surgical approach**	**CI side**	**Follow up**	**Test method**
Wang et al. (26)	Retrospective	China	34	4–15 years	LVAS 18 Normal CT 16	RW/ Extended RW	Unilateral	9 months	cVEMP oVEMP caloric test vHIT
Koyama et al. (7)	Not specified	Japan	73	10.58 years	Genetic mutation 31 Virus infection 11 Syndrome 5 Inner ear malformations 5 Other 2 Unknown 19	RW/ Extended RW /Cochleostomy	Bilateral	33 months	cVEMP
Wang et al. (27)	Retrospective	China	16	5–18 years	EVA 16	RW/ Extended RW	Unilateral	12 months	Cvemp oVEMP
Guan et al. (28)	Retrospective	China	22	6–17 years	Hereditary 5 Drug–induced 1 viral infection 5 Unknown 11	RW	Unilateral and bilateral	1 month	cVEMP oVEMP caloric test vHIT
Wolter et al. (29)	Not specified	Canada	52	6–18 years	Usher syndrome 7 Meningitis 4 Cochleovestibular anomaly 3 Unknown etiology 3 CMV 1 Normal 34	Not specified	Bilateral	Not specified	BOT−2
Reynard et al. (30)	Retrospective	France	15	1.67–6 years	Mondini malformation 3 Pendred syndrome 2 LC malformation 1 Enlarged IAC 1 Nomal CT 8	RW	Bilateral	6 months	cVEMP vHIT
Wolter et al. (31)	Prospective	Canada	26	6–18 years	Usher syndrome 7 Unknown 5 Meningitis 3 Cochleovestibular anomalies 2 Nomal 10	Not specified	Bilateral	Not specified	BOT−2
Li et al. (32)	Prospective	China	35	3–18 years	EVA 14 Normal CT 21	RW	Unilateral	5 days, 1 month, 2 months	cVEMP oVEMP
Cozma et al. (33)	Prospective	Romania	80	4.35 years	Not specified	RW/Cochleostomy	Unilateral and bilateral	3 months	cVEMP
Gupta et al. (34)	Prospective	India	25	3–7 years	Profound SNHL 23 Severe SNHL 2	Cochleostomy	Not specified	6 weeks	Caloric test
Ajalloueyan et al. (35)	Prospective	Iran	27	1–4.67 years	Not specified	RW	Unilateral	6–8 weeks	Cvemp caloric test
Hazzaa et al. (36)	Not specified	Egypt	40	3–14 years	Heredofamilial 16 Unknown 13 Heredofamilial + Postfebrile 3 Heredofamilial+ Neonatal insult 2 Waardenberg syndrome 2 Ototoxicity 2 Perinatal insult 1	Not specified	Not specified	1 months 6 months	cVEMP oVEMP
Devroede et al. (15)	Retrospective	Belgium	26	6.75 years	Clinical syndrome 7 Genetic mutations 7 Postmeningitis 1 CMV infection 1 Auditory neuropathy spectrum disorder 2 Unknown 8	Cochleostomy	Sequentially implanted	3 months	Cvemp caloric test
Xu et al. (37)	Prospective	China	31	3–12 years	Not specified	Cochleostomy	Unilateral	4 weeks	Cvemp oVEMP
Psillas et al. (38)	Prospective	Greece	10	1.5–4 years	Congenital idiopathic deafness without inner ear dysplasia or syndrome 10	Cochleostomy	Unilateral	10 days, 6 months	cVEMP
Eustaquio et al. (39)	Observational	USA	64	8.16 years	Nonimplanted 26 Unilateral implant 12 Bilateral implants 26	Not specified	Unilateral and bilateral	Not specified	BOT−2
Licameli et al. (40)	Prospective	Boston	19	8 years	Not specified	Not specified	Unilateral	4–6 weeks	cVEMP
Cushing et al. (41)	Observational	Canada	56	4–17 years	Cochlear implant 41 Normal 14	Not specified	Unilateral	4.8 years	BOT−2
Jin et al. (42)	Not specified	Japan	24	2–14 years	Not specified	Not specified	Not specified	Not specified	cVEMP
Jin et al. (43)	Not specified	Japan	12	2–7 years	Mondini 2 one branch of vestibulocochlear nerve 1 EVA 1 Normal 8	Not specified	Not specified	Not specified	cVEMP

BOT−2, Bruininks–Oseretsky Test 2; cVEMP, cervical vestibular–evoked myogenic potential; CMV, cytomegalovirus; EVA, enlarged vestibular aqueduct syndrome; Extended, extended RW; IAC, Internal auditory canal; LSC, lateral semicircular canal; LVAS, large vestibular aqueduct syndrome; oVEMP, ocular vestibular–evoked myogenic potential; RW, round window; SNHL, sensorineural hearing loss; vHIT, video head impulse test.

### Results of the otolith function tests

**Figure 2:**
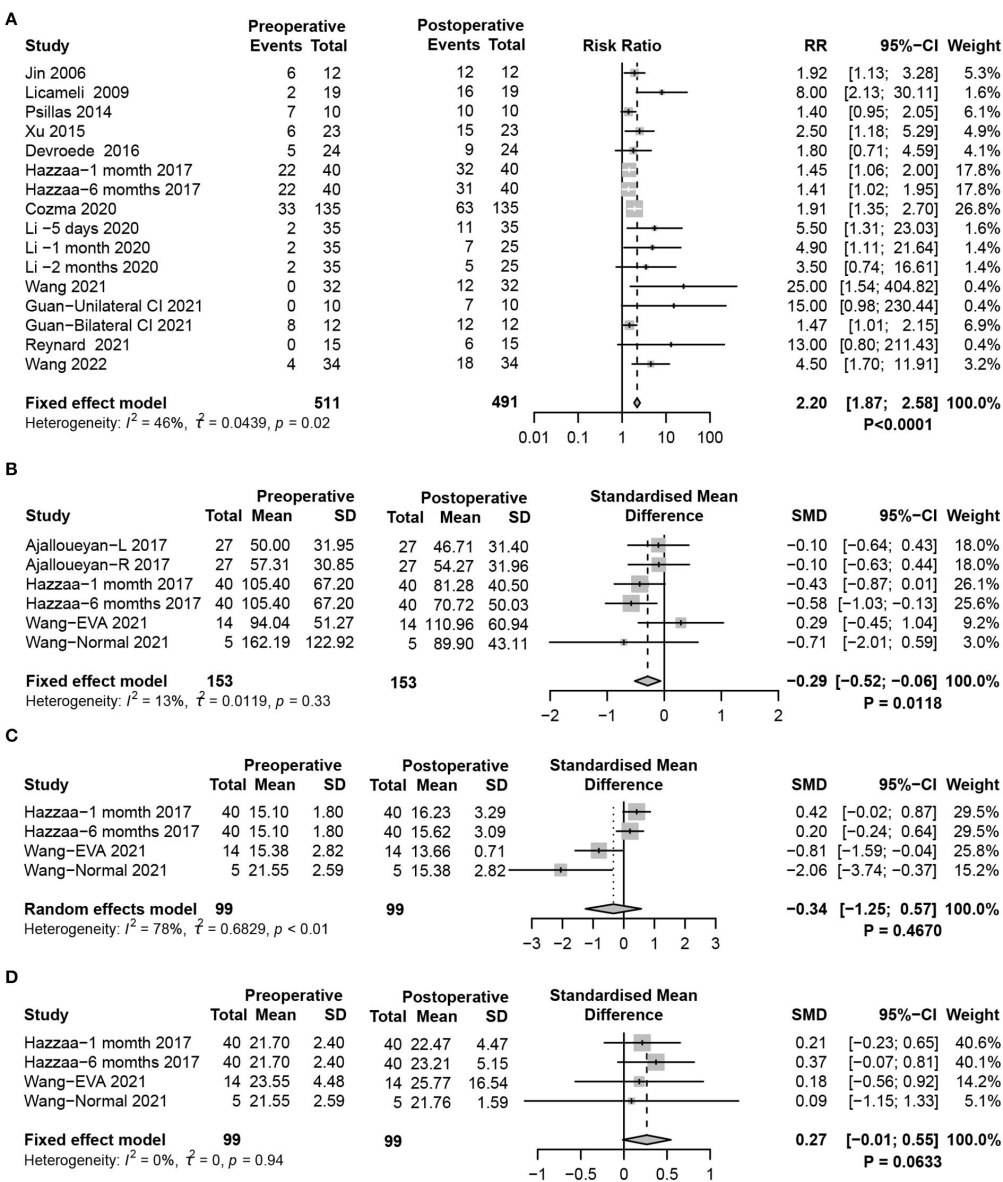
Forest plots showing the saccular function test results between pre- and post-surgery groups. **(A)** Response to the cVEMP test. **(B–D)** Response of cVEMP parameters including **(B)** P1-N1 amplitude, **(C)** P1 latency, and **(D)** N1 latency. Study, included studies for Research on meta-analysis; Preoperative, results of vestibular function test before operation; Postoperative, results of vestibular function test after operation; Events, number of people with abnormal vestibular function test results; Total, total number of patients in the study; Mean, arithmetic mean; SD, standard deviation; RR, relative risk; 95%-CI, 95% confidence interval; SMD, standardized mean difference; Weight, weight of each study in statistics.

cVEMP, which is produced from the saccule and transmitted through the ipsilateral inferior vestibular nerve, induces the ipsilateral sternocleidomastoid to produce an inhibitory potential. The cVEMP test is an established technique for evaluating saccular function. The present meta-analysis defined weak or disappearing cVEMP response as otolith organ dysfunction. Statistical analysis demonstrated significant impairment of saccular function after CIM in children (fixed-effects model, RR = 2.20, 95% CI = 1.87, 2.58, *P* < 0.0001) (Figure 2A). In addition, cVEMP response parameters showed significantly reduced P1-N1 amplitudes in the postoperative period (fixed-effects model, SMD = −0.29, 95% CI = −0.52, −0.06, *P* = 0.0118), while no significant changes in P1 (random-effects model, SMD = −0.34, 95% CI = −1.25, 0.57, *P* = 0.4670) and N1 latencies (fixed-effects model, SMD = 0.27, 95% CI = −0.01, 0.54, *P* = 0.0633) were observed (Figures 2B–D).

**Figure 3:**
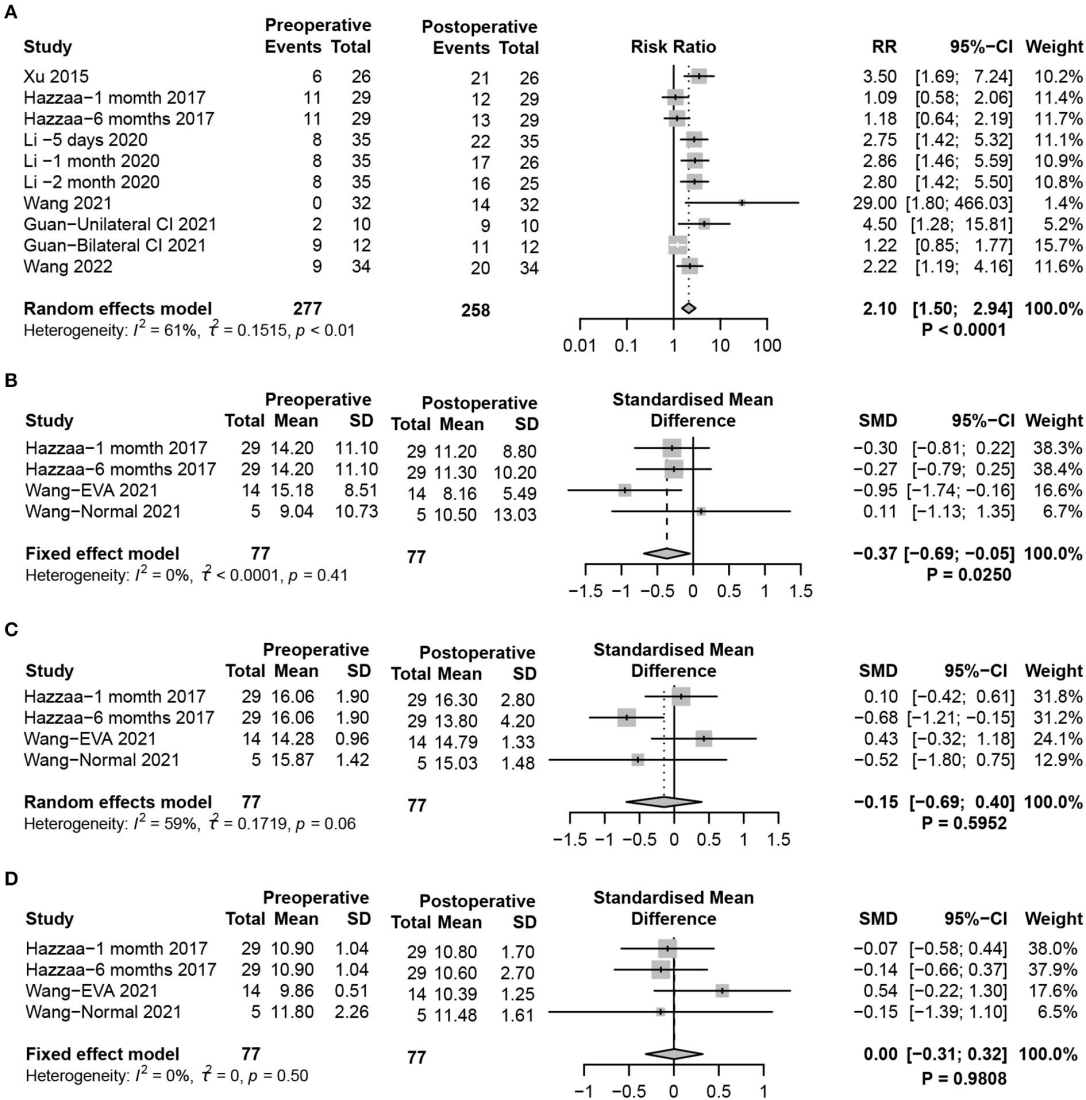
Forest plots showing utricle function test results between pre- and post-surgery groups. **(A)** Response to the oVEMP test. **(B–D)** Response of oVEMP parameters including **(B)** P1-N1 amplitude, **(C)** P1 latency, and **(D)** N1 latency. Study, included studies for Research on meta-analysis; Preoperative, results of vestibular function test before operation; Postoperative, results of vestibular function test after operation; Events, number of people with abnormal vestibular function test results; Total, total number of patients in the study; Mean, arithmetic mean; SD, standard deviation; RR, relative risk; 95%-CI, 95% confidence interval; SMD, standardized mean difference; Weight, weight of each study in statistics.

oVEMP, mainly induced by the utricle, is transmitted through the superior vestibular nerve to induce the excitatory potential of the contralateral musculus obliquus inferior bulbi. oVEMP reflects the function of the utricle-superior vestibular nerve reflex pathway. Similar to the results of cVEMP, significant damage to utricle function in postoperative pediatric patients was found (random-effects model, RR = 2.10, 95% CI = 1.50, 2.94, *P* < 0.0001) (Figure 3A). Additionally, by analyzing the response parameters of oVEMP, a significant weakening of the P1-N2 amplitude after CIM in children was identified (fixed-effects model, SMD = −0.37, 95% CI = −0.69, −0.05, *P* = 0.0250). There were no significant differences in P1 (random-effects model, SMD = −0.15, 95% CI = −0.69, 0.40, *P* = 0.5952) and N1 (fixed-effects model, SMD = 0.00, 95% CI = −0.31, 0.32, *P* = 0.9808) latencies in oVEMP after CIM (Figures 3B–D).

### Results of the tests for semicircular canal function

**Figure 4:**
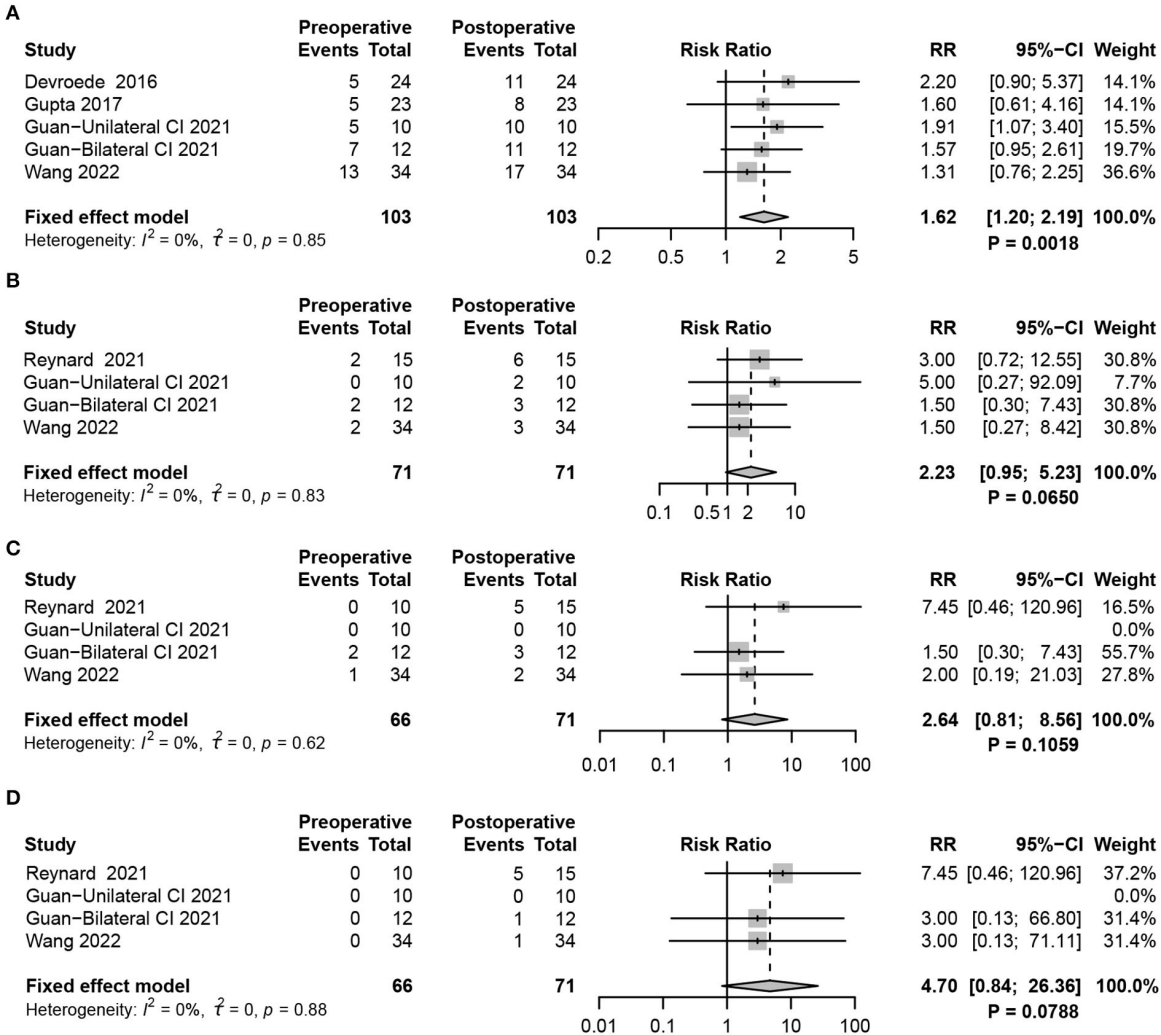
Forest plots showing semicircular canal function test results between pre- and post-surgery groups. **(A)** Response to the caloric test. **(B–D)** vHIT, including **(B)** HSC **(C)** PSC, and **(D)** ASC function tests. Study, included studies for Research on meta-analysis; Preoperative, results of vestibular function test before operation; Postoperative, results of vestibular function test after operation; Events, number of people with abnormal vestibular function test results; Total, total number of patients in the study; RR, relative risk; 95%-CI, 95% confidence interval; Weight, weight of each study in statistics.

The caloric test detects the vestibulo-ocular reflex (VOR), which reflects the function of the left and right horizontal semicircular canals (HSCs), evaluating the status of vestibular function at ultralow frequencies. The results of the caloric test analysis are shown in the forest plot (Figure 4A). By comparing the collection of nystagmus pre- and postoperatively, statistical analysis revealed a significant effect of CIM on the caloric test results. The increased risk of abnormal reactions in the caloric test demonstrated that HSC function was seriously damaged after CIM in children (fixed-effects model, RR = 1.62, 95% CI = 1.20, 2.19, *P* = 0.0018).

In recent years, vHIT has become a comprehensive examination method to assess the function of the semicircular canals [HSC, posterior semicircular canal (PSC), and anterior semicircular canal (ASC)]. In contrast to the caloric test, vHIT completes the examination of three pairs of semicircular canals to evaluate vestibular function status at high frequencies. VOR gain was used to determine the function of the semicircular canals (VOR <0.8 considers HSC dysfunction, while the dysfunction of PSC and ASC was VOR <0.7). The fixed-effects meta-analysis did not indicate any significant differences after CIM in VOR gain detection for HSC and PSC, demonstrating that normal function might be preserved in HSC (RR = 2.23, 95% CI = 0.95, 5.23, *P* = 0.0650), PSC (RR = 2.64, 95% CI = 0.81, 8.56, *P* = 0.1059), and ASC (RR = 4.70, 95% CI = 0.84, 26.36,*P* = 0.0788 (Figures 4B–D).

### Results of the balance function test

**Figure 5:**
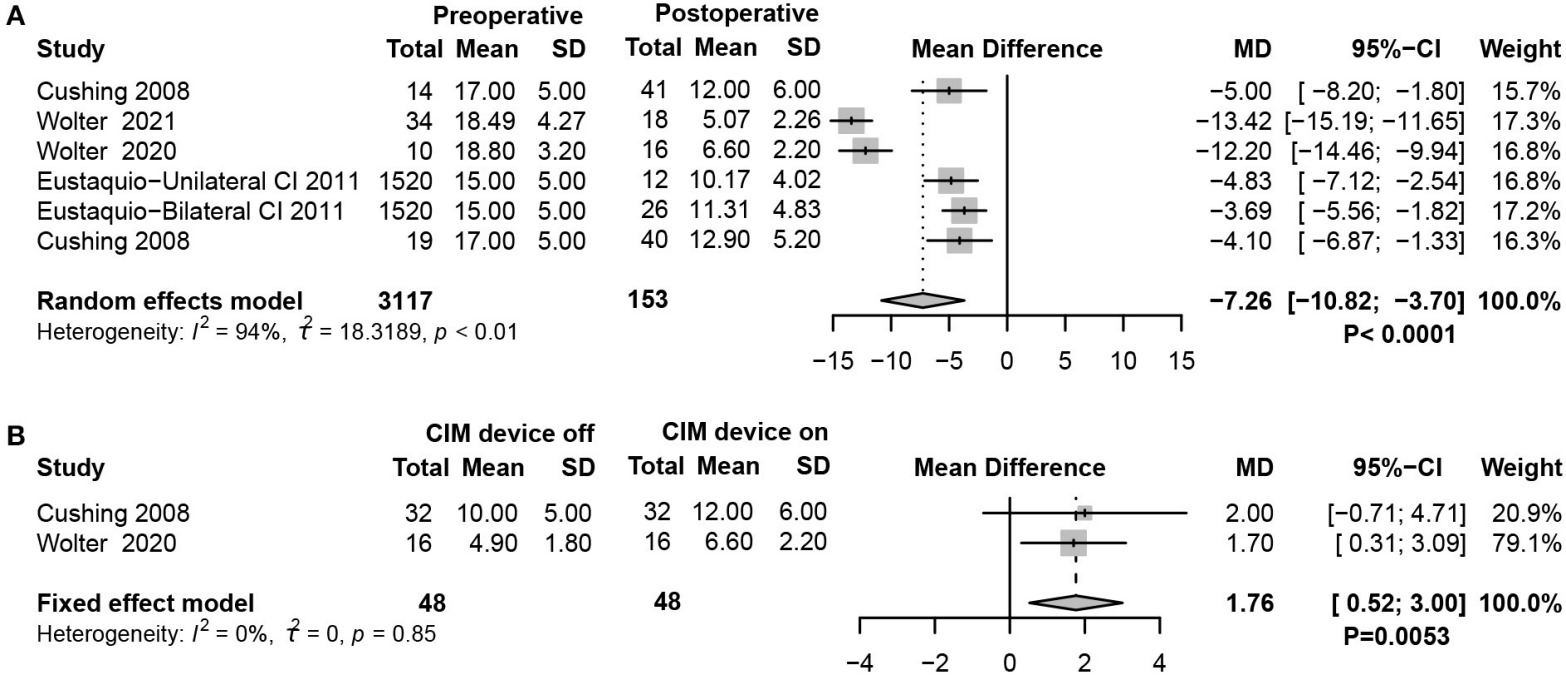
Forest plots showing balance function test results between pre- and post-surgery groups. **(A)** BOT-2 test scores. **(B)** Comparison of the balance function between CIM devices switched on and off. Study, included studies for Research on meta-analysis; Preoperative, results of vestibular function test before operation; Postoperative, results of vestibular function test after operation; CIM device off, postoperative results of vestibular function test with CIM devices off; CIM device on, postoperative results of vestibular function test with CIM devices on; Total, total number of patients in the study; Mean, arithmetic mean; SD, standard deviation; MD, mean difference; 95%-CI, 95% confidence interval; Weight, weight of each study in statistics.

The balance subtest of BOT-2 evaluates static and dynamic balance functions by scoring nine balance tasks, with higher scores indicating better overall static and dynamic balance. The results revealed that balance was significantly worse in children with SNHL who received CIM than in children with typical hearing (random-effects model, MD = −7.26, 95% CI = −10.82, −3.70, *P* < 0.0001) (Figure 5A). Interestingly, when the CIM device was on, the BOT-2 score slightly improved compared with when the CIM device was off, which suggested that providing sound inputs through implants positively affects balance in children with SNHL (fixed-effects model, MD = 1.76, 95% CI = 0.52, 3.00, *P* = 0.0053) (Figure 5B).

### Factors affecting changes in vestibular function

**Figure 6:**
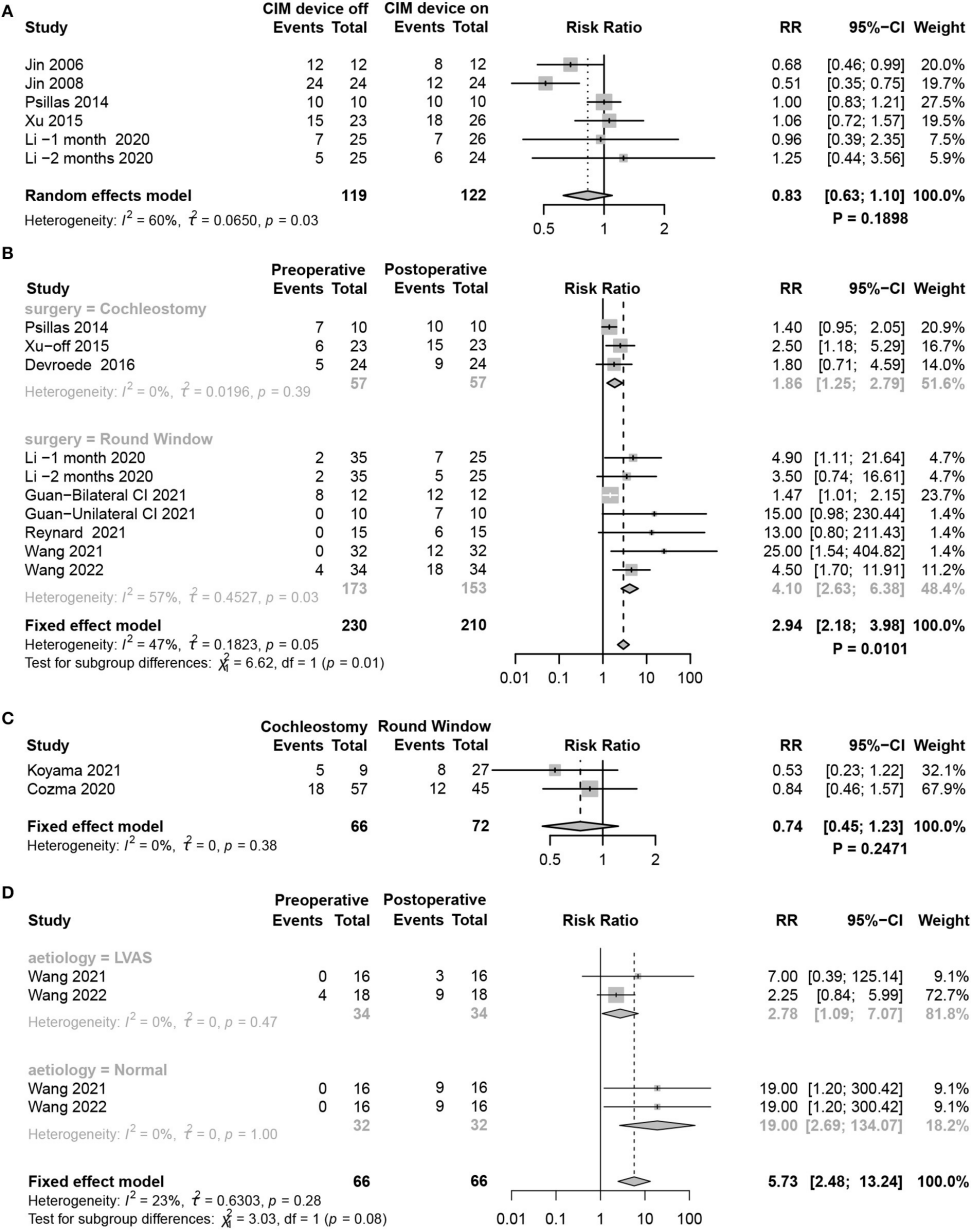
Forest plots showing factors affecting vestibular function changes. **(A)** cVEMP test comparing CIM devices on and off. **(B)** Subgroup analysis of patients using RW and cochleostomy. **(C)** Comparison of the effect of RW and cochleostomy on vestibular function. **(D)** Subgroup analysis comparing the effect of LAVS and normal patients on vestibular function. Study, included studies for Research on meta-analysis; CIM device off, postoperative results of vestibular function test with CIM devices off; CIM device on, postoperative results of vestibular function test with CIM devices on; Preoperative, results of vestibular function test before operation; Postoperative, results of vestibular function test after operation; Cochleostomy, cochleotomy implantation group; Round Window, round window implantation group; Events, number of people with abnormal vestibular function test results; Total, total number of patients in the study; RR, relative risk; 95%-CI, 95 confidence interval; Weight, weight of each study in statistics.

Considering the benefit of maintaining balance in children with CIM devices, the meta-analysis compared the results of tests assessing objective vestibular function using cVEMP between CIM devices on and off. However, no significant difference was found between the two groups (random-effects model, RR = 0.83, 95% CI = 0.63, 1.10, *P* = 0.1898) (Figure 6A).

RW and cochleostomy are the two most common surgical approaches for CIM port electrode insertion. Although both caused vestibular dysfunction, the meta-analysis revealed that children receiving RW acquired more severe damage (*P* = 0.0101) (Figure 6B). While directly contrasting the effect of vestibular function between the two methods, no statistically significant difference was found (fixed-effects model, RR = 0.74, 95% CI = 0.45, 1.23, *P* = 0.2471) (Figure 6C).

Vestibular dysfunction occurred in about half of the children with profound SNHL before CIM. The likelihood was highly dependent on their individual etiologies. In the absence of specific aetiological data from the included literature, we only compared whether a difference in the degree of vestibular dysfunction would occur between children with LVAS and normal children after CIM. Although the abnormality rate of cVEMP after CIM was higher in normal patients than in those with LAVS, subgroup analysis showed no statistically significant difference between the two groups (*P* = 0.0819) (Figure 6D).

### Risk of bias across studies

The risk of bias when comparing the studies was deemed low. No concerns were identified regarding the selective reporting of data because patients in the reviewed studies were generally accounted for in the results.

## Discussion

### Background

Cochlear implantation may also lead to vestibular dysfunction. In studies involving adults, Hansel et al. (23) observed a significantly increased postoperative risk of imbalance, vertigo, and falls as well as a significant impairment of otolithic organs and canal function. Similar results were observed in pediatric patients. A significant reduction in cVEMP response was observed after CIM in children (24). Inadequate labyrinth protection is considered a major cause of vestibular symptoms (44). Specifically, several potential mechanisms of surgical injuries include serous labyrinthitis induced by the opening of the membranous labyrinth (45, 46), permanent damage in the endolymphatic system caused by the direct injury caused by electrode array insertion in the implantation process (47), mechanical disruption of inner ear structures (48–53), or temporary lymph flow obstruction caused by blood, fibrous tissue, and bone powder (54).

Due to the challenges in accomplishing vestibular tests in the pediatric population, few studies, especially systematic and comprehensive analyses, have reported vestibular function changes pre- and postoperatively in children who receive CIM. Therefore, to measure the specific impact of CIM surgery on vestibular function in children, our meta-analysis confirmed that the vestibular function of the pediatric population was significantly damaged after CIM by comparing the function of the otoliths, semicircular canals, and balance.

### Otolith function after CIM

Previous evidence has reported that the abnormal response or parameters of the VEMPs are present in pediatric patients with CIM (15, 26, 28, 30, 32, 33, 36–38, 40, 43). The statistical analysis of the VEMPs' responses showed that the abnormal response of VEMPs significantly increased after CIM, which proved that CIM could potentially cause damage to both utricle and saccular functions in pediatric patients. Due to the lack of literature on the results of VEMP parameters, we only found lower amplitudes in the postoperative cVEMP and oVEMP tests (27, 35, 36). Only two studies have reported specific P1 and NI latency data, and inconsistent results were presented. Comprehensive analysis showed that the difference was not statistically significant in the P1 and NI latencies of cVEMP and oVEMP (27, 36).

Because the saccule is closer to the electrode insertion pathway anatomically, some studies have considered that the saccule is more susceptible to damage than the utricle (55, 56). However, some studies have reported divergent results. Li et al. (32) showed significant differences between the response rates of cVEMP and oVEMP after CIM, highlighting that the utricle may be more vulnerable to surgery. In addition, no significant difference between the response rates of cVEMP and oVEMP after CIM was found by Xu et al. (37). Therefore, we compared the meta-analysis results of cVEMP and oVEMP to verify which one is more easily damaged, and the outcome demonstrated no significant difference between the two tests. Further in-depth studies with larger sample sizes are needed to confirm this conclusion.

### Semicircular canals function after CIM

In addition to otoliths, vestibular organs include three pairs of semicircular canals. To comprehensively evaluate vestibular function in pediatric patients after surgery, we evaluated all three pairs of semicircular canal function under high-frequency impulse stimulation by integrating the vHIT results. Meanwhile, a caloric test assessed HSC function under a low-frequency stimulus. Practically, these vestibular function tests are quite difficult to perform in children. Increased abnormal rates from pre- to post-implantation in caloric tests, but not in vHIT, suggested that the detection of calorie tests was more sensitive than vHIT in pediatric patients. Similar results from Nassif et al. (57) showed no significant difference in HSC VOR gain between the implanted and non-implanted-implanted sides in unilaterally implanted children; the function on both sides was similar to that in children with normal hearing. The deterioration risk ratio was increased in HSC tested by caloric testing (RR=1.62, *P* = 0.0018), while HSC tested by vHIT showed no significant difference. The vHIT and caloric tests measured two extreme frequency ranges of the HSC VOR. The vHIT uses a physiological stimulus with higher testing frequencies (>1 Hz), close to the physiological stimuli of daily life, whereas the caloric test applies a non-physiological stimulus (<0.003 Hz), and the parallel recovery processes in vestibular function between the two tests were different (58). The other evidence, attempting to validate the caloric test compared with vHIT, discovered that HSC VOR gain in high-frequency stimulus results is abnormal only when vestibular impairment on caloric testing of the semicircular canals is higher than 40% (59). These two measures should be performed together to comprehensively assess semicircular canal function.

### Balance function after CIM

Although CIM improves hearing and speech perception in SNHL, this technique can also cause balance deficiencies or increase existing balance dysfunction (60). BOT-2 has become the most widely standardized method for assessing motor proficiency. It is a clinical test battery comprising several subtests, one of which was designed to evaluate the overall balance function (61). As expected, with lower BOT-2 scores, balance ability was significantly worse in children with SNHL requiring CIM than in typically developing children with hearing impairment. Nevertheless, when pediatric patients received any sound with their implant device, the rising BOT-2 score indicated that the postural balance function slightly improved. Postural stability can also be measured using posturography and center-of-pressure variation as a function of time (62–64). The same conclusion was reached even with other evaluation methods (65). Stabilizing postural control requires the optimal integration of information from somatosensory, visual, vestibular, and other sensory systems (hearing, tactile, etc.) (66). Thus, auditory information can improve postural stability in children with balance disorders (31, 41).

### Factors affecting changes in vestibular function

We also compared the changes in vestibular function when the cochlear implant device was turned on or off. Some research results indicated that although the saccular function was damaged before surgery, the VEMP response was elicited again upon activation of the CIM device (32, 42, 43). For instance, the study demonstrated that 11 out of 12 children showed no response in cVEMPs when the cochlear implant was turned off, whereas four children had reproducible cVEMPs when switched on (43). A comparison of the cVEMP parameters found that lower thresholds on the implanted sides and wider amplitudes on the contralateral side were achieved with the CIM device (32). The possible reason is that galvanic stimulation from the CIM device may evoke a myogenic response in the sternocleidomastoid muscle (67, 68). However, other studies have not supported this conclusion. In the study by Psillas et al. (38), the VEMPs remained absent irrespective of device activation. Therefore, we conducted a summary analysis of relevant studies and found no significant difference in vestibular function changes between CIM devices on and off. Evidently, our findings were based on a small sample, and there was great variability among these studies. Further research is necessary for an in-depth understanding of vestibular changes with CIM devices on and off.

The surgical approach is an important consideration affecting the preservation of the vestibular neurosensory epithelium and cochlea. RW and cochleostomy are widely used to enrich the intracochlear space. Clinically and histopathologically, previous studies have identified that RW is better than cochleostomy, especially in effectively preserving vestibular functions (43, 69–72). For example, Todt et al. (73) reported hypofunction of postoperative cVEMP in 13% of patients who underwent RW, while 50% underwent cochleostomy. The reason port electrode insertion by cochleostomy induces a risk of vestibular loss is probably due to the drilling, which produces mechanical and thermal aggression.

Additionally, the bony drilling residue may penetrate the inner ear and even produce ossifications (33). However, electrode insertions through the RW membrane resulted in deep atraumatic insertions into the scala tympani. Thus, previous studies suggested that to preserve vestibular functions to the greatest extent, RW is the better technique (74). In our study, we calculated the RR to directly compare the differences in vestibular function damage between the two surgical methods. Compared with cochleostomy, Koyama et al. (7) and Cozma et al. (33) reported that the risk of vestibular loss was reduced by 47 and 16%, respectively, when performing RW. Nevertheless, no significant difference was observed. A subgroup analysis involving the indirect comparison of the results of different studies showed the opposite results; compared with cochleostomy, RW increased the risk of vestibular dysfunction. We inferred that although cochleostomy produces greater surgical trauma and bone scarring, the RW membrane is closer to the saccule anatomically. Furthermore, previous studies were mainly based on adult patients, and pediatric implantation surgeries in the included cohorts were performed by different surgeons using distinct techniques. Consequently, the degree of vestibular function damage caused by RW and cochleostomy in pediatric patients is difficult to define; further verification is needed to clarify this conclusion. Follow-up research should focus on this aspect through a comprehensive assessment of hearing and vestibular function in pediatric patients before surgery, carefully confirming the differences in anatomical structures of different patients and determining the eligible surgical method.

The likelihood of vestibular dysfunction is highly dependent on etiology, with meningitis and cochleovestibular anomalies having the highest rates of severe dysfunction (75). LAVS is the most common abnormal radiologic finding in pediatric patients with SNHL (76), and it has a high rate of vestibular pathology (77). Comparing the extent of vestibular dysfunction between children with LVAS and normal children after CIM revealed a significant increase in the overall abnormality rate of the VEMP from pre- to post-CIM in normal patients but no significant change in children with LVAS. This could be because, in children with LVAS, the pressure generated during electrode insertion could be released through the enlarged vestibular aqueduct or into the endolymphatic fluid, resulting in less impairment (26). Besides the vestibular dysfunction, peripheral mechanical changes were considered. However, the subgroup analysis found no statistical significance between the two groups, most likely due to insufficient sample size and corresponding cohort studies. The effect of etiology on vestibular function is significant, and our future work will collect more relevant data for statistical analyses. We propose that more attention should be paid to the detailed assessment of pre- and postoperative vestibular function in pediatric patients with the underlying condition of vestibular dysfunction.

### Comprehensive evaluation of vestibular function before and after CIM

In addition, about half of pediatric cochlear implant candidates already suffer from vestibular deficits, and 51% of cochlear implants result in changes in existing preoperative vestibular function. Given the high prevalence of vestibular dysfunction after CIM in our meta-analysis, any implantation should be preceded by functional testing of the semicircular canals and otolith. Preoperative vestibular function testing is not only useful to check for vestibular dysfunction associated with congenital SNHL, but it can also determine the side of CIM. If only one functional vestibule is present, the least functional vestibule should be selected as the side for the CIM to limit the likelihood of bilateral vestibular loss, except in cases where audiological or anatomical criteria are important (40). Similarly, a vestibular assessment should be performed before bilateral simultaneous or sequential implantation to prevent complete bilateral vestibular areflexia and its potential consequences.

The postoperative test is also indispensable. It is better suited to comprehensively assessing the changes in vestibular function. The vestibular function should be evaluated not only when the pediatric patients show symptoms related to vestibular disorders, such as dizziness or vertigo, but in all patients that underwent CIM. It should be kept in mind that the subjects are children who may have difficulty describing their symptoms clearly. If vestibular function tests were only conducted after the onset of obvious symptoms, this would lead to an increased diagnosis rate and delayed treatment. We conclude that CIM can lead to vestibular dysfunction. Thus, assessing vestibular function after surgery is vital to assure early diagnosis and treatment.

To sum up, we should not only pay attention to the degree of hearing restoration after CIM but also to the vestibular dysfunction in pediatric patients to detect and treat it in time.

### Limitations

Most studies classified abnormal VEMP response as hyporeflexia or areflexia. Only some studies reported specific VEMP response parameters. Additionally, the CIM device state, etiologies of SNHL, and the surgical approach may affect the vestibular function of the pediatric population. Most children are unable to accurately describe their symptoms. This makes it difficult to assess their subjective perception of dizziness or vertigo. The Dizziness Handicap Inventory is often used to evaluate the quality of life of adults, but this questionnaire is not suited for children. Consequently, we did not analyze the occurrence of dizziness and vertigo in pediatric patients after CIM. We will further collect the latest articles in the future, which also validates our analysis results.

## Conclusions

The present study confirmed that the disappearance and impairment of cVEMP, oVEMP, and caloric response could be observed after CIM in pediatric patients, reflecting damage to the utricle, saccule, and HSC caused by CIM. In addition, the patients' balance ability significantly decreased after the operation. All the evidence indicates that vestibular dysfunction is common in pediatric patients with SNHL after CIM, suggesting that apart from audiological or anatomical criteria being the main concern of CIM in pediatric patients, vestibular function should be considered.

## Data availability statement

The original contributions presented in the study are included in the article/Supplementary material, further inquiries can be directed to the corresponding authors.

## Author contributions

QW designed the study, screened the literature, conducted statistical analyses, and drafted the manuscript. QZ assisted in drafting the protocol, collecting data and processing, and editing the manuscript. YZ and ZC assessed the quality of inclusion research. QX and SL performed data extraction and literature screening. YX, XW, and X-DX prepared the figures and revised the manuscript. JL, YJ, JY, and QZ critically evaluated the manuscript. All authors reviewed and approved the final version of the manuscript.

## Funding

This research was funded by the National Natural Science Foundation of China (Grant Nos. 82171137, 81970891, 81860189, and 81970876), Clinical Research Plan of SHDC (SHDC2022CRD013), the Key International Cooperation Project of Shaanxi Province (Grant No. 2020KWZ-019), and the Science and Technology Project of the Shanghai Science and Technology Commission (Grant No. 21S31900600).

## Conflict of interest

The authors declare that the research was conducted in the absence of any commercial or financial relationships that could be construed as a potential conflict of interest.

## Publisher's note

All claims expressed in this article are solely those of the authors and do not necessarily represent those of their affiliated organizations, or those of the publisher, the editors and the reviewers. Any product that may be evaluated in this article, or claim that may be made by its manufacturer, is not guaranteed or endorsed by the publisher.

## Abbreviations

ASC: anterior semicircular canal
BOT-2: Bruininks-Oseretsky Test of Motor Proficiency 2
CI: confidence interval
CIM: cochlear implantation
CMV: cytomegalovirus
cVEMP: cervical vestibular-evoked myogenic potential
EVA: enlarged vestibular aqueduct syndrome
Extended: extended RW
HSC: horizontal semicircular canal
IAC: Internal auditory canal
LVAS: large vestibular aqueduct syndrome
MD: mean diffusivity
oVEMP: ocular vestibular-evoked myogenic potential
PSC: posterior semicircular canal
RR: relative risk
RW: round window
SMD: standardized mean difference
SNHL: sensorineural hearing loss
VEMP: vestibular-evoked myogenic potential
vHIT: video head impulse test
and VOR: vestibulo-ocular reflex.
